# Automatic end‐to‐end VMAT treatment planning for rectal cancers

**DOI:** 10.1002/acm2.14259

**Published:** 2024-02-05

**Authors:** Kai Huang, Christine Chung, Ethan B. Ludmir, Lifei Zhang, Constance A. Owens, Jean Gumma‐De La Vega, Jack Duryea, Yao Zhao, Xinru Chen, David Fuentes, Carlos E. Cardenas, Tina Marie Briere, Sam Beddar, Laurence E. Court, Prajnan Das

**Affiliations:** ^1^ The University of Texas MD Anderson Cancer Center UTHealth Houston Graduate School of Biomedical Sciences Houston Texas USA; ^2^ Department of Radiation Physics The University of Texas MD Anderson Cancer Center Houston Texas USA; ^3^ Department of Gastrointestinal Radiation Oncology The University of Texas MD Anderson Cancer Center Houston Texas USA; ^4^ Department of Imaging Physics The University of Texas MD Anderson Cancer Center Houston Texas USA; ^5^ Department of Radiation Oncology The University of Alabama at Birmingham Birmingham Alabama USA

**Keywords:** automatic contouring, automatic treatment planning, rectal cancer, rectal radiotherapy, segmentation, VMAT

## Abstract

**Background:**

The treatment planning process from segmentation to producing a deliverable plan is time‐consuming and labor‐intensive. Existing solutions automate the segmentation and planning processes individually. The feasibility of combining auto‐segmentation and auto‐planning for volumetric modulated arc therapy (VMAT) for rectal cancers in an end‐to‐end process is not clear.

**Purpose:**

To create and clinically evaluate a complete end‐to‐end process for auto‐segmentation and auto‐planning of VMAT for rectal cancer requiring only the gross tumor volume contour and a CT scan as inputs.

**Methods:**

Patient scans and data were retrospectively selected from our institutional records for patients treated for malignant neoplasm of the rectum. We trained, validated, and tested deep learning auto‐segmentation models using nnU‐Net architecture for clinical target volume (CTV), bowel bag, large bowel, small bowel, total bowel, femurs, bladder, bone marrow, and female and male genitalia. For the CTV, we identified 174 patients with clinically drawn CTVs. We used data for 18 patients for all structures other than the CTV. The structures were contoured under the guidance of and reviewed by a gastrointestinal (GI) radiation oncologist. The predicted results for CTV in 35 patients and organs at risk (OAR) in six patients were scored by the GI radiation oncologist using a five‐point Likert scale. For auto‐planning, a RapidPlan knowledge‐based planning solution was modeled for VMAT delivery with a prescription of 25 Gy in five fractions. The model was trained and tested on 20 and 34 patients, respectively. The resulting plans were scored by two GI radiation oncologists using a five‐point Likert scale. Finally, the end‐to‐end pipeline was evaluated on 16 patients, and the resulting plans were scored by two GI radiation oncologists.

**Results:**

In 31 of 35 patients, CTV contours were clinically acceptable without necessary modifications. The CTV achieved a Dice similarity coefficient of 0.85 (±0.05) and 95% Hausdorff distance of 15.25 (±5.59) mm. All OAR contours were clinically acceptable without edits, except for large and small bowel which were challenging to differentiate. However, contours for total, large, and small bowel were clinically acceptable. The two physicians accepted 100% and 91% of the auto‐plans. For the end‐to‐end pipeline, the two physicians accepted 88% and 62% of the auto‐plans.

**Conclusions:**

This study demonstrated that the VMAT treatment planning technique for rectal cancer can be automated to generate clinically acceptable and safe plans with minimal human interventions.

## 1 INTRODUCTION

Rectal cancer is a common type of cancer, with an incidence of more than 732 000 and mortality of more than 339 000 globally each year.^1^ The standard of care for rectal cancer includes preoperative radiotherapy. Volumetric modulated arc therapy (VMAT) is used for the treatment of rectal cancers for its potential to reduce toxicity to organs at risk (OARs) surrounding the tumor site by reducing radiation dose delivered to these OARs.^2^ The radiation dose can be administered using conventionally fractionated radiotherapy (long‐course radiotherapy) or hypofractionated radiotherapy (short‐course radiotherapy).^3^ Long‐course radiotherapy uses doses of 1.8–2 Gy per fraction for 25–28 daily fractions, along with concurrent chemotherapy. Short‐course radiotherapy uses 5 Gy per fraction for five fractions. The VMAT technique is often recommended for treating patients using the short‐course fractionation scheme or when a treatment plan based on the 3D conformal technique, infers a heightened risk for treatment toxicity.^2^ However, creating a patient‐specific treatment plan is a challenging process that requires a highly specialized workforce and is labor‐intensive and time‐consuming.^4^ The treatment planning process can be divided into two major components: structure segmentation and planning. Structure segmentation requires the delineation of the target volume and all OARs for each patient on all slices of simulation CT scans. The planning process is dependent on the accurate delineation of structures, especially the target volumes. Therefore, automation of the treatment planning process requires the automation of structure segmentation and planning individually, and also as an end‐to‐end process to examine the tolerance of contouring errors propagating to the subsequent planning step.

Recent advances in deep learning have allowed efficient and accurate segmentation of structures in medical images.^5^ Development of the nnU‐Net method by Isensee et al. provided a streamlined three‐dimensional (3D) segmentation solution for 3D biomedical images that surpassed many existing solutions in segmentation competitions.^6^ For rectal cancers specifically, Men et al.^7^ have applied a 2D deep dilated convolutional neural network (CNN) to auto‐segment OARs and clinical target volume (CTV) on CT scans. However, the clinical acceptability of the contour predictions was unclear. Here, we start by presenting segmentations of CTV and OARs with extensive evaluations of their clinical acceptability.

Knowledge‐based planning leverages a set of previously used radiotherapy plans to statistically estimate the dose‐volume histogram (DVH) of a given patient.^8^ Commercial vendors offer knowledge‐based planning in their treatment planning systems. RapidPlan from Varian Medical Systems, for example, has been used in auto‐planning for different body sites and achieved plans that were non‐inferior to manually created plans in multiple disease sites, including the prostate, prostatic fossa, lung, and head and neck.^9^, ^10^ Although knowledge‐based planning training provides DVH predictions and some weights, extensive fine‐tuning, including the addition of planning structures and additional planning objectives, is necessary in order to create a model that works for the majority of patients without needing additional per‐patient iterations.^10^ Olanrewaju et al. showed that RapidPlan can be used to achieve high‐quality automated plans consistently, even for challenging disease sites such as head‐and‐neck regions.^10^ Previous study has also explored producing clinically acceptable VMAT plans automatically for cervical cancers based on automatically segmented contours.^11^

The goal of this project was to assess whether the entire VMAT planning process, including both segmentation and plan optimization, could be automated to achieve high clinical acceptability without user interventions for rectal cancers, using a 3D CNN architecture and an automated RapidPlan program. The study examined the quality of plans generated based on auto‐segmentations and evaluated holistically the feasibility and clinical impacts of using an end‐to‐end system in producing consistent and clinically acceptable plans, all based on extensive physician evaluations.

## 2 METHODS

### 2.1 Data

This study was performed using CT scans and medical records from patients with malignant neoplasm of rectum treated at The University of Texas MD Anderson Cancer Center. Patients were selected by searching the database for treatments coded with C20 billing code. This study was approved by the Institutional Review Board, which waived informed consent because of the retrospective nature of the study. Table 1 describes the number of training and testing data used for each experiment and their corresponding clinical characteristics such as patient setup and sex. The prone patients’ data used in auto‐segmentation of OARs and CTV, and auto‐planning may have overlaps. However, the data used in the end‐to‐end test were previously unseen and completely independent of all the data used in auto‐segmentation and auto‐planning processes.

**TABLE 1:** Number of patients data used for each experiment specified by training and testing and the clinical characteristics in terms of male or female, and prone or supine patient setup.

	Number of patients (train/test)	Comment
Auto‐segmentation: OARs	18 (12/6)	All prone, 18 patients for each OAR, 36 patients in total with 18 females and 18 males
Auto‐segmentation: CTV	174 (139/35)	All prone
Auto‐planning	54 (20/34)	20 supine, 34 prone
End‐to‐end	16	Independent data, prone, eight females, eight males

### 2.2 Auto‐segmentation

In this study, we used simulation CT scans and clinically‐approved target volumes from patients retrospectively. We included patients who were simulated in a prone, arms‐up position with a belly board for preoperative treatment of rectal cancer. We trained, validated, and tested deep learning auto‐segmentation models using nnU‐Net architecture^12^ for a total of nine structures. The structures were the pelvic CTV and the following OARs: bowel bag, large bowel, small bowel, total bowel (large and small bowel combined), femurs, bladder, bone marrow, and female or male genitalia.

**FIGURE 1:**
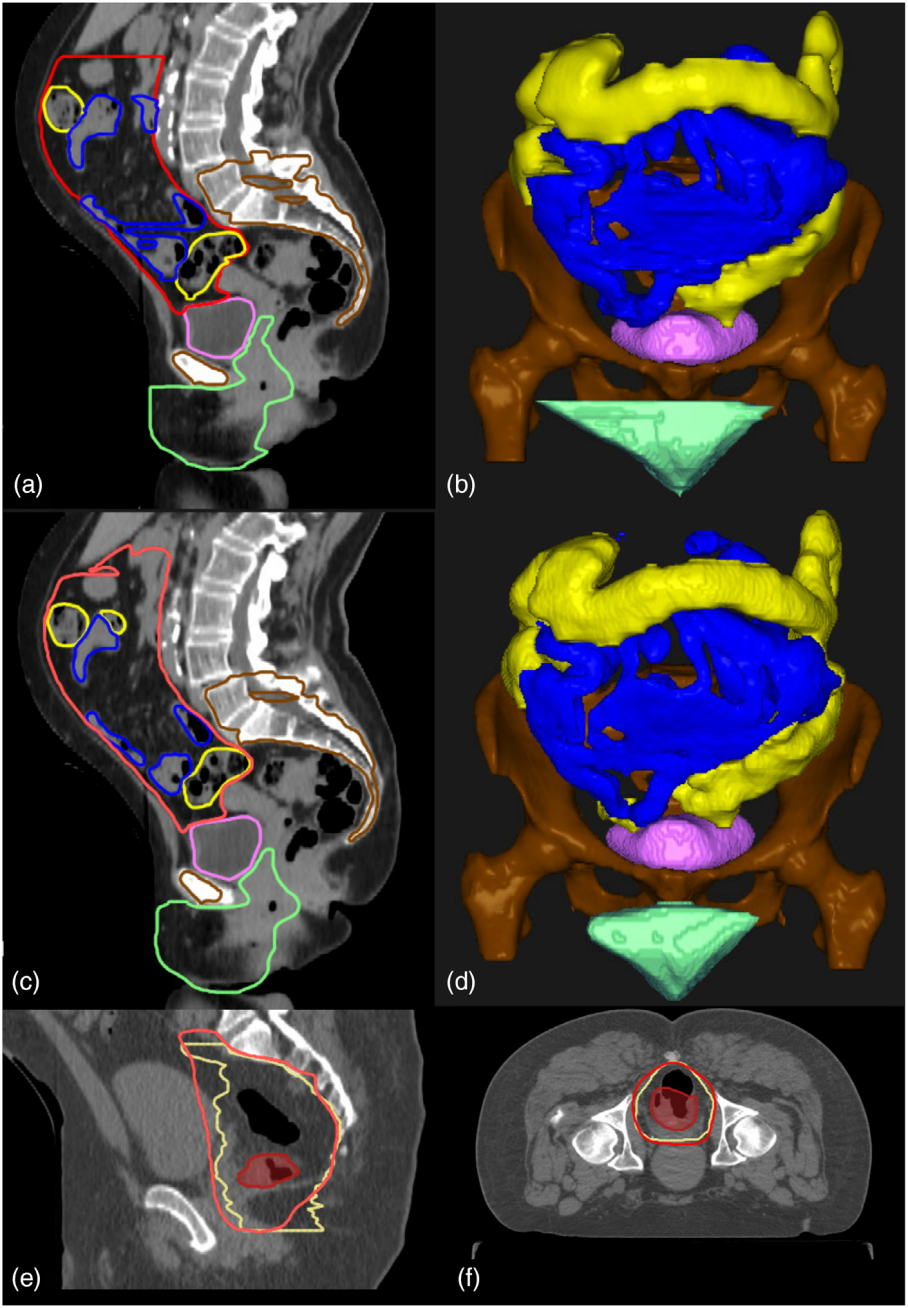
Examples of contours for ROIs. (a, b) Ground‐truth contours as determined by a GI radiation oncologist. (a) Sagittal 2D view. (b) Coronal 3D view. (c, d) Auto‐segmentation predicted contours (a, c Sagittal 2D view). (d) Coronal 3D view. Bowel bag is in red, large bowel is in yellow, small bowel is in blue, bone marrow is in brown, bladder is in pink, and female genitalia is in green. (e, f) Clinical GTV in shaded red, clinical CTV in yellow, and predicted CTV in red. (e) Sagittal view. (f) Axial view.

For OARs, we used CT data from 36 patients (18 female, 18 male). The scans were acquired between 2018 and 2021. Patients who had undergone colostomy prior to radiotherapy were excluded from the dataset due to significant changes to the anatomy of the bowels caused by surgical procedures. All the non‐sex‐dependent OARs were drawn on the 18 female patients. The 18 male patients only had the male genitalia drawn. Therefore, each OAR structure had 18 contours from 18 patients. Though these patient numbers were relatively low, a previous study has shown that clinically acceptable predictions can be achieved with consistent data curation using low numbers of patient scans.^13^ The slice thicknesses of the CT scans were either 2, 2.5, or 3 mm. The pixel spacing of the CT scans ranged from 0.98 to 1.3 mm. The OAR contours were manually drawn under the guidance of an expert gastrointestinal (GI) radiation oncologist; these were considered the “ground‐truth” contours. The quality of training data, measured largely by consistency, is critical to obtaining an effective model; therefore, all the contours for OARs were evaluated slice by slice and approved by the radiation oncologist before training and testing. Figure 1 gives examples of contours for some OARs used in training. The bowel bag contours started inferiorly from the most inferior bowel loop. Contours for the large bowel did not include the rectum in order to not extend the OAR substantially into target volumes. The contours for the bowel bag, large bowel, and small bowel extended superiorly to the end of the scan or the end of the structure, whichever was most inferior. The contour for femurs included the femoral head and femur down to the lesser trochanter. The female genitalia structure included the external genitals and vagina. The male genitalia structure included the scrotum and penis. Bladder was drawn from the base to the dome. The bone marrow structure included the pelvic girdle, femurs, and L5 vertebral body.

For the CTV, we retrospectively identified 174 patients with clinically drawn CTV and gross tumor volume (GTV). Patients with external iliac node or inguinal node coverage in the CTV were excluded from the dataset. The slice thicknesses of the CT scans were either 1, 2, 2.5, or 3 mm. The pixel spacing of the CT scans ranged from 0.98 to 1.3 mm. An example of a CTV with GTV is shown in Figure 1(e, f). The CTV covered the GTV and the mesorectal, presacral, and internal iliac nodes. The inferior border of the CTV was at either the pelvic floor or 2 cm inferior to the distal extent of the GTV. The superior border of the CTV went up to the bifurcation of the common iliac artery or the L5/S1 intervertebral body location.

To reduce time spent in predicting contours for deployment purposes, we trained one single‐class model for total bowel, two multiclass models for other OARs, and one single‐class model for CTV with clinical primary rectal tumor GTV and involved nodal GTV (GTVn) as inputs. The GTV inputs were provided to the CTV model as binary masks with a value of one inside the GTV region and zero elsewhere. For each OAR structure, training and testing used data from 12 and six patients, respectively. For CTV, training and testing used data from 139 and 35 patients, respectively. All models were trained using 3D full resolution U‐Net with default configuration, pre‐processing, and data augmentation techniques.^6^ The optimizer was stochastic gradient descent. The loss function was the sum of the cross‐entropy and Dice similarity coefficients loss.^12^ Five‐fold cross‐validation was used for all model training, and the testing was performed on the testing dataset with five‐fold ensemble.

To evaluate the contours, we calculated Dice similarity coefficients, Hausdorff distances, 95% Hausdorff distances, and mean surface distances. The resulting contours from each model were evaluated by an expert GI radiation oncologist based on a five‐point Likert scale (Table 2).

**TABLE 2:** Five‐point scale for evaluating the quality of predicted field apertures and plans.

Score	Acceptability	Description
5	Acceptable—Use as‐is	Clinically acceptable, could be used for treatment without change
4	Acceptable—Minor edits that are not necessary	Stylistic differences, but not clinically important; the current contours/plans are acceptable
3	Unacceptable—Minor edits that are necessary	Edits that are clinically important, but it is more efficient to edit the automatically generated contours/plans than to start from scratch
2	Unacceptable—Major edits	Edits that are required to ensure appropriate treatment and sufficiently significant that the user would prefer to start from scratch
1	Unacceptable—Unusable	Automatically generated contours/plans are so bad that they are unusable (i.e., wrong body area, outside confines of body, etc.)

### 2.3 Auto‐planning

The next step after segmenting the contours is planning. To automatically create plans, a RapidPlan knowledge‐based planning tool provided by Varian Eclipse (version 15.5) was modeled by using retrospective plans from patients who had received VMAT delivery with a prescription of 25 Gy in five fractions in a supine setup. The model was trained and tested on 20 and 34 patients, respectively. The patients received VMAT between 2015 and 2021. The 20 patients in the training set were chosen because of the consistency in their respective plans. The model used 6‐MV beam quality. Of the total 20 patient plans for training, five had three arcs, 14 had two arcs, and one had four arcs. All 20 training patients had been set up in a supine position. The model was tested on 15 supine and 19 prone cases as patients may be treated in either position. The model used three arcs having gantry rotation from 182 degrees to 178 degrees clockwise, 178 degrees to 182 degrees counter clockwise, and 182 degrees to 178 degrees clockwise, with 10, 350, and 90 degrees collimator rotations. The optimization objectives of the model were created to meet the goals and constraints used in our clinic. The objectives for planning target volume (PTV) included V100% > 95%, V95% > 99%, V105% < 10%, and maximum dose (Dmax) < 120%. The dose objectives for normal tissues were the following: For small bowel, the objectives were Dmax < 27.8 Gy, V25 Gy < 65 cm^3^, V22.2 Gy < 100 cm^3^, and V19.5 Gy < 180 cm^3^. For femurs, the objectives were Dmax < 27.8 Gy, V25 Gy < 25%, and V22.2 Gy < 40%. For bladder structure, the objectives were Dmax < 27.8 Gy, V25 Gy < 15%, and V22.2 Gy < 40%. For bone marrow, the objectives were V20 Gy < 35% and V15 Gy < 50%. Clinical contours and 16 planning structures created based on clinical contours were used for training and testing of the model. Clinical target contours included the CTV and PTV. Clinical OARs used included the bladder, small bowel, left and right femurs, bone marrow, and genitals. The planning structures used were created using algebraic operations on clinical structures for the purpose of ensuring suppressing dose to the OARs. All the bowel structures were combined into one for planning purposes. The PTV was a 0.5‐cm expansion of the CTV. The collimator setting was set at 0.5 cm beyond the PTV. To increase the model's stability and robustness, the objectives' priority was adjusted to result in a final model with as many generated objectives as possible.

Plan optimization was then automated through an in‐house program in C# language based on the Varian Eclipse API. The program automatically creates PTV and planning structures given clinical OARs and CTV, performs DVH estimation, and optimizes for and calculates dose.

To evaluate the validity of the model, all the testing plans were scored by two expert GI radiation oncologists using a five‐point Likert scale (Table 2). The values of the testing plans for each dose objective were reported and compared against the objectives’ values in box plots.

### 2.4 End‐to‐end evaluation

To test the full integrated process, we used retrospective CT scans and clinical primary rectal tumor GTV and involved nodal GTVn from 16 previously unseen patients (eight female, eight male). All of them had been set up in prone position with belly boards and had no previous colostomy or use of a vaginal dilator. Two GI radiation oncologists reviewed the predicted contours for all structures, including CTV and OARs, then scored the CTV structure and the final plan on the five‐point Likert scale (Table 2). The average time to create an automatic plan with the given structures was measured and reported. The values of the 16 plans for each dose objective were reported and compared against the objectives’ values from both our clinic and the consensus objectives from the Veteran Affairs Radiation Oncology Quality Surveillance Program and American Society for Radiation Oncology Expert Panel (consensus objectives).^14^

## 3 RESULTS

### 3.1 Auto‐segmentation

Table 3 shows the auto‐segmentation analysis results in terms of mean and standard deviation (SD) of Dice similarity coefficient, mean surface distance, Hausdorff distance, and 95% Hausdorff distance for each structure. Rigid bony structures (bone marrow and femurs) achieved superior prediction results. Bladder, bone marrow, total bowel, bowel bag, femurs, and male genitalia had mean Dice similarity coefficients over 0.9. CTV, female genitalia, large bowel, and small bowel achieved Dice coefficients between 0.83 and 0.89. Larger structures had larger values in distance measures. Bowel structures (total bowel, large bowel, small bowel, and bowel bag) had mean 95% Hausdorff distances between 5.56 and 26.57 mm. The scores were better for total bowel than for large or small bowel. The values shown in Table 3 corroborated the physician scores in Table 4; for bone marrow and femurs, all contours were scored as five. Bladder, female, and male genitalia, total bowel, and bowel bag were also scored as 100% clinically acceptable (scores four or five). Large and small bowel contours achieved 33% clinical acceptability, with two of six contours being clinically acceptable and the rest needing minor necessary edits. CTV achieved 89% clinical acceptability, with four of 35 plans deemed as needing minor necessary edits and the rest deemed to have stylistic differences that were not clinically necessary.

**TABLE 3:** Auto‐segmentation results. Mean and standard deviation of Dice similarity coefficient, mean surface distance, Hausdorff distance, and 95% Hausdorff distance for each structure (CTV, N = 35; OARs, N = 6).

	Dice similarity coefficient		Mean surface distance (mm)		Hausdorff distance (mm)		95% Hausdorff distance (mm)	
ROI	Mean	SD	Mean	SD	Mean	SD	Mean	SD
CTV	0.85	0.05	4.33	1.84	26.66	9.18	15.25	5.59
Femurs	0.97	0.01	0.56	0.28	7.27	3.65	2.5	1.36
Bone marrow	0.96	0.01	0.38	0.1	11.43	3.57	1.94	1.25
Bladder	0.95	0.07	0.6	0.55	4.97	3.11	2.32	2.29
Total bowel	0.92	0.04	0.82	0.27	42.24	20.13	5.56	5.52
Bowel bag	0.94	0.04	2.4	1.2	35.75	17.41	11.35	6.22
Small bowel	0.87	0.05	2.34	1.3	47.24	13.52	12.93	6.97
Large bowel	0.83	0.11	3.91	3.1	60.28	26.96	26.57	16.58
Female genitalia	0.89	0.03	1.29	0.57	20.51	11.74	6.46	2.8
Male genitalia	0.94	0.03	1.11	0.51	13.75	6.06	4.21	2.65

Abbreviation: CTV, Clinical target volume.

**TABLE 4:** Physician scoring of auto‐segmentation results for each ROI (CTV, *N* = 35; OARs, *N* = 6).

	Counts per score					Clinical acceptability (%)	
ROI	5	4	3	2	1	Acceptable without edits	Acceptable with minor edits
CTV	0	31	4	0	0	89%	11%
Femurs	6	0	0	0	0	100%	0%
Bone marrow	6	0	0	0	0	100%	0%
Bladder	5	1	0	0	0	100%	0%
Total bowel	3	3	0	0	0	100%	0%
Bowel bag	2	4	0	0	0	100%	0%
Small bowel	0	2	4	0	0	33%	67%
Large bowel	0	2	4	0	0	33%	67%
Female genitalia	1	5	0	0	0	100%	0%
Male genitalia	3	3	0	0	0	100%	0%

Abbreviations: CTV, clinical target volume; ROI, region of interest.

**FIGURE 2:**
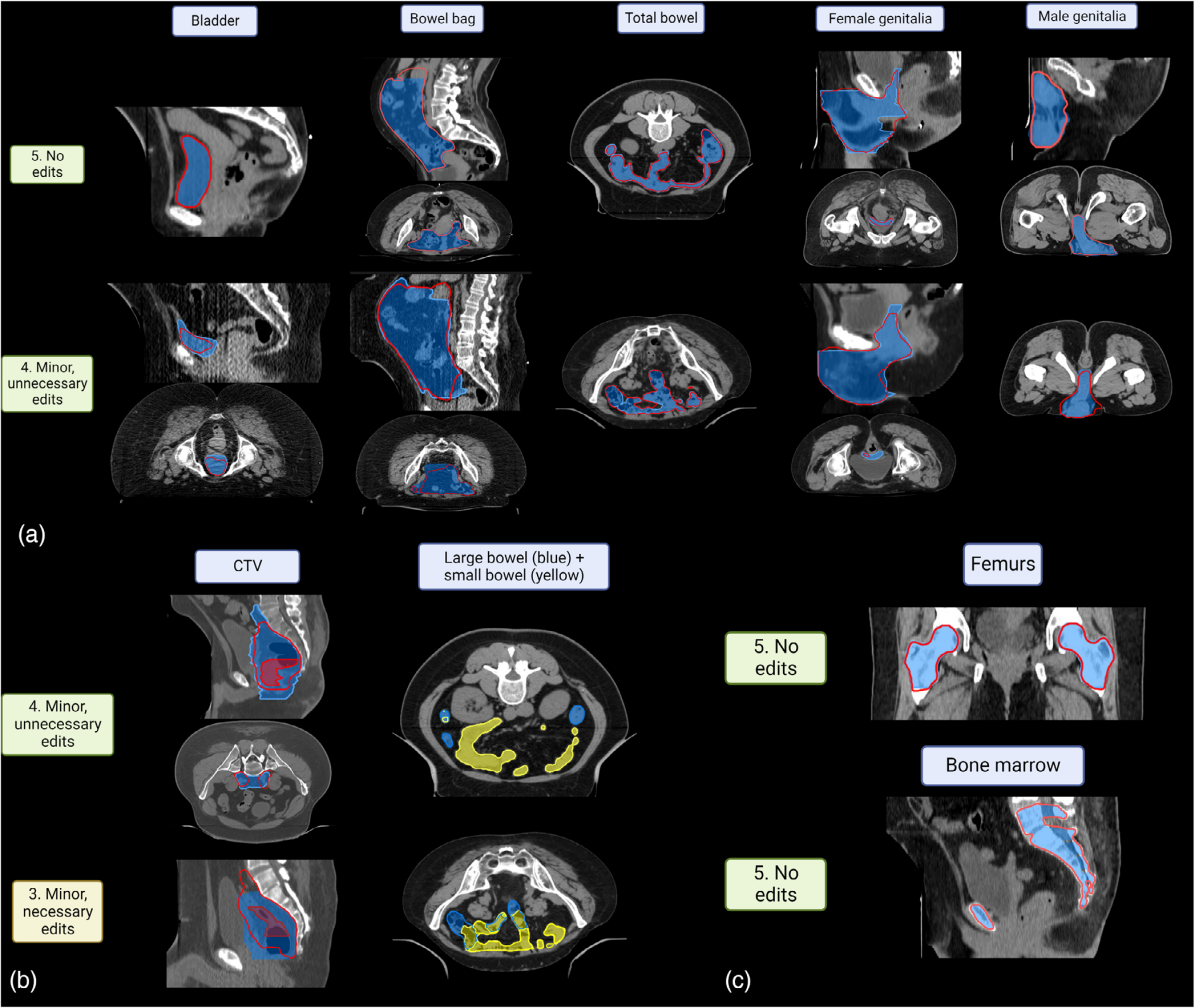
Examples of testing (red) and ground‐truth (shaded in blue) contours for each structure, with physician scores. (a) Bladder, bowel bag, total bowel, and female and male genitalia contours scored as five or four. (b) CTV and large and small bowel scored as four or three. The ground‐truth contours for the large and small bowels were shaded in blue and yellow, respectively. The predicted contours for the large and small bowels were displayed as blue and yellow contour outlines, respectively. (c) Femurs and bone marrow scored as five.

Figure 1 shows an example of predicted CTV and OARs. Figure 2 shows typical examples of each structure by the scores assigned. The bladder, bowel bag, bowel, female genitalia, and male genitalia structures received scores of five and four (Figure 2a). The reasons for structures scoring less than five varied. In the example in which the bladder was scored as four, the contour did not extend posteriorly sufficiently to cover the bladder structure, most likely because of the excessive streaking artifacts in the CT scan. In the example bowel bag that was scored as four, the contour was disconnected on the shown axial slice. Contours for bowels were scored as four for reasons such as small and disconnected contours being drawn, and small, insufficient contours in some areas. Female genitalia contours were scored as four because the most superior slices did not sufficiently include the cervix. The example male genitalia contour was scored as four because of incomplete coverage on the right side of the patient shown on the axial slice. Figure 2(b) shows CTV and large and small bowels with examples of contours scored as four or three. The CTV example scored as four had loops on the most superior slice that were disconnected. All of the cases in which CTV was scored as three were because of insufficient coverage for either the GTVn or GTV or in one case, an overextension of CTV on one side anteriorly. For the example CTV scored as three, the model did not include sufficient slices inferior to the GTV in the CTV prediction because of the excessive gas volume in the rectum. As for bowels, depending on the texture of the bowel loops, sometimes large bowels were mistaken as small bowels and vice versa. The contours in which fewer mistakes happened and appeared further away from the target were scored as four, and the rest were scored as three.

### 3.2 Auto‐planning

**FIGURE 3:**
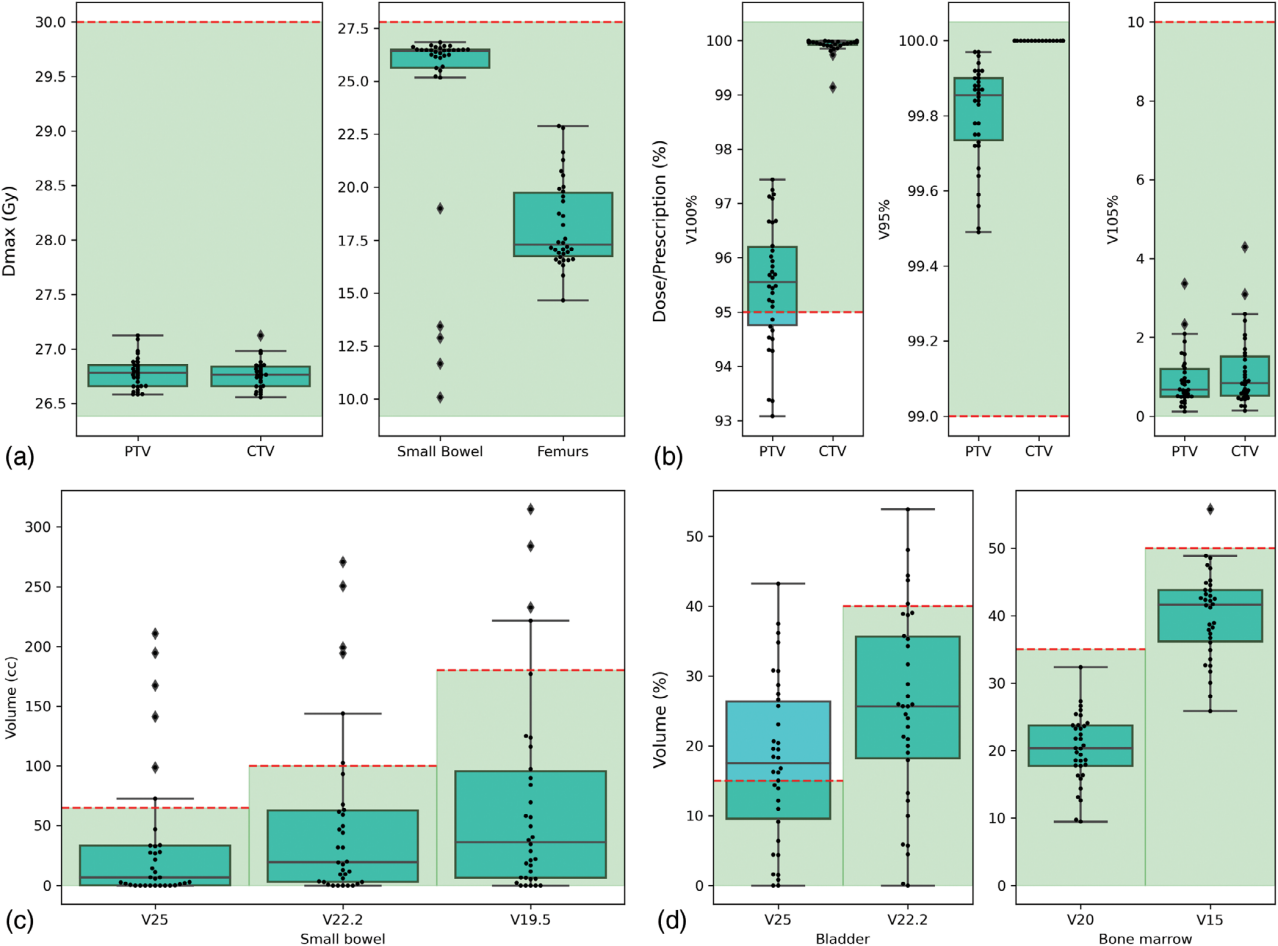
Boxplots of dose metrics for the 34 auto plans created in the auto‐planning testing step using our clinics’ dose objectives. The red dashed lines indicate objective values, and the green shaded regions indicate all the values satisfying the corresponding objectives.

The auto‐plans’ values for ROI objectives used in our clinics are summarized for the 34 testing cases in Figure 3. The average V100% values for PTV and CTV were 95.53% ± 1.13% and 99.93% ± 0.15%, respectively. The average minimum dose (Dmin) values for PTV and CTV were 22 ± 0.62 Gy and 24.64 ± 0.38 Gy, respectively. In terms of hot spots, the average Dmax received by PTV was 26.78 ± 0.14 Gy, which is 107% of the prescription dose shown in Figure 3(a). No V105% exceeded 10% of PTV nor 10% of CTV. In terms of OAR goals, the volume goals for femurs were not included in the figure because both V25 and V22.2 Gy for femurs were equal to 0%. Bladder objectives were the most challenging to reach, with 17 plans achieving the V25 Gy < 15% goal and 28 plans achieving V22.2 Gy < 40%. This was because portions of the bladder volumes often fell within the PTV. Out of 34 plans, 28 plans met the small bowel constraints completely. Out of the six plans that did not meet all the small bowel goals, two were scored as three and the rest were scored as four by one of the physicians (Table 5). Between the two physicians, the average clinical acceptability of the plans was 96%, with one physician scoring all the plans as clinically acceptable and the other scoring 91% of the plans as clinically acceptable without edits. Reasons for plans being scored as three included too high of a dose to small bowel, and PTV coverage being insufficient. Reasons for plans being scored as four included isodose lines breaking up, causing small cold spots, and that more sparing would be preferred for bowel structures or bladder.

**TABLE 5:** Physician scoring for each auto‐plan for validating the RapidPlan model (*N* = 34).

	Counts per score					Clinical acceptability (%)	
Physician	5	4	3	2	1	Acceptable without edits	Acceptable with minor edits
1	31	3	0	0	0	100%	0%
2	16	15	3	0	0	91%	9%

### 3.3 End‐to‐end evaluation

**FIGURE 4:**
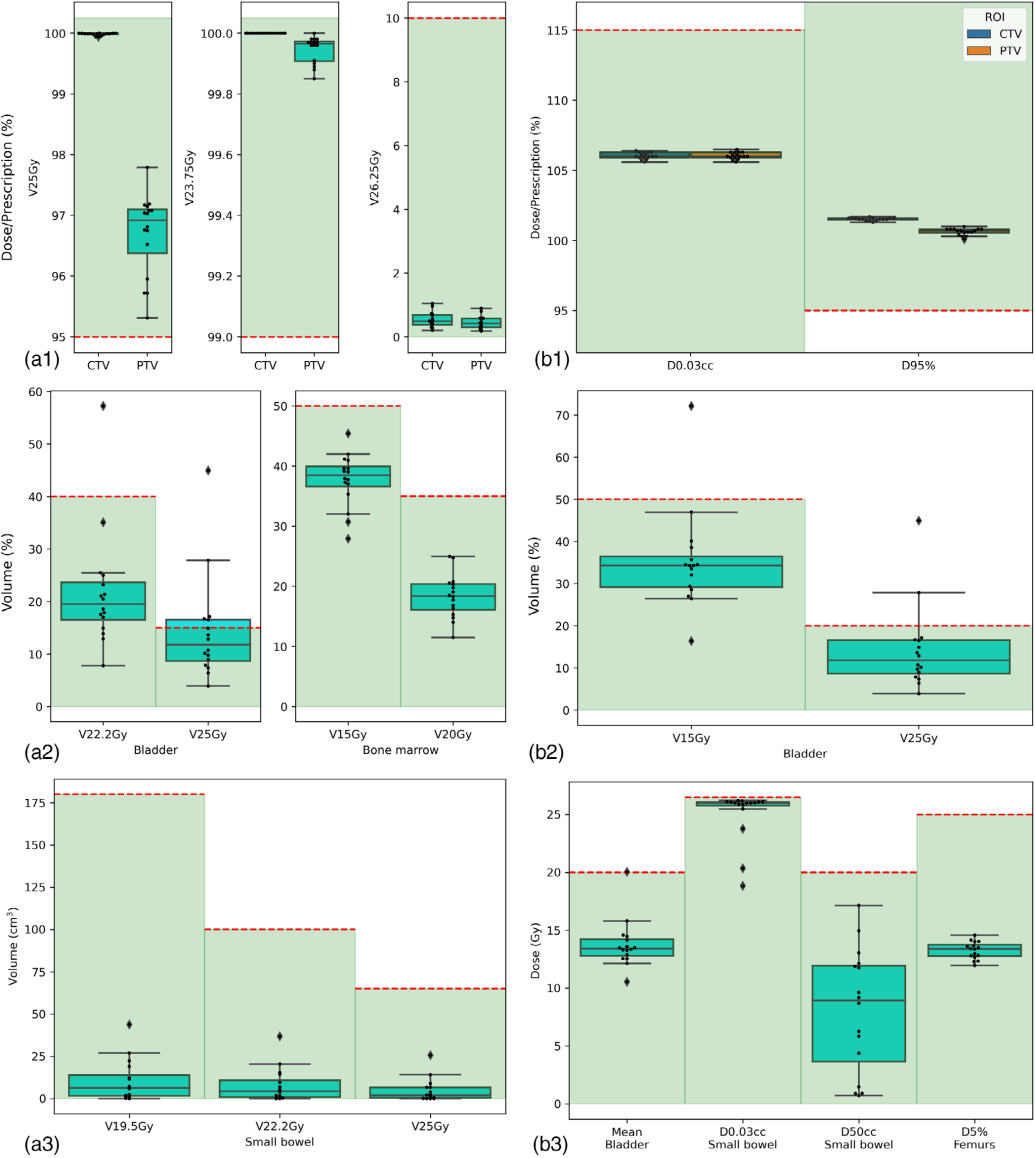
Box plots of structures satisfying the objective values for 16 end‐to‐end evaluations based on (a) institutional objectives and (b) ASTRO consensus dose constraints.^14^ (a1) Dose percentages of predicted CTV and PTV. (a2) Volume percentages of bladder and bone marrow structures. (a3) Volumes for small bowel structure at various dose levels. (b1) CTV and PTV dose objectives. (b2) Volume percentage constraints of bladder structure. (b3) Dose objectives of bladder, small bowel, and femurs. The red dashed lines indicate objective values, and the green shaded regions indicate all the values satisfying the corresponding objectives.

For the 16 cases in whom we tested the full integrated process, the average (± SD) time to automatically produce the plans given the structures was 33.4 ± 6.1 min. Figure 4 shows the box plots of the DVH values for each structure with respect to both our institutional planning objectives and the ASTRO consensus objectives.^14^ The average (±SD) Dmax values for CTV and PTV were both 26.6 ± 0.1 Gy. The average (±SD) Dmin values for CTV and PTV were 24.8 ± 0.1 Gy and 22.7 ± 0.3 Gy, respectively. The average (±SD) Dmax value for femurs was 17.9 ± 1.5 Gy. None of the plans had Dmax reaching 27.8 Gy, which was the Dmax objective for all OARs. The female genitalia received an average (±SD) V15 Gy of 15.3% ± 9.2% and V20 Gy of 13.5% ± 8.5%. All of the plans satisfied all of the consensus objectives except for only one plan that did not meet the bladder objectives. Due to the proximity to the target volumes, the bladder would have received a dose higher than the objectives; however, this did not affect the clinical acceptability of the plan.

The physicians evaluated the CTV predicted automatically based on clinical GTV and GTVn for each patient. As shown in Table 6, for physician one, 10 CTV contours were acceptable without any edits, six CTV contours required minor necessary edits, and no CTV contours required major necessary edits. For physician 2, 14 CTV contours were acceptable without any edits, two CTV contour required minor necessary edits, and no CTV contour required major necessary edits. The reasons for CTV contours being scored as requiring edits were insufficient coverage in inferior slices covering the anal canal, insufficient coverage anteriorly bilaterally in the obturator nodal basin, insufficient coverage to the sacrum, and breakups of contours in the most superior and inferior slices. In one case, the GTV input only covered the tumor volume but not the entire rectum lumen on slices where there was tumor. This caused the predicted CTV contour not to cover the anterior portion of the rectum adequately. On average, the acceptability percentage for CTVs without edits was 75%.

**TABLE 6:** Physician scoring of each end‐to‐end plan and the predicted CTVs associated with the plans (*N* = 16).

		Counts per score					Clinical acceptability (%)	
Physician		5	4	3	2	1	Acceptable without edits	Acceptable with minor edits
1	CTV	1	9	6	0	0	62%	38%
	Plans	9	1	6	0	0	62%	38%
2	CTV	1	13	2	0	0	88%	12%
	Plans	11	3	2	0	0	88%	12%

Following the CTV reviews, the radiation oncologists scored the end‐to‐end plans for each patient (Table 6). All the plans in which the CTV needed edits were deemed to require replanning, which would be an automatic process after manual edits and was therefore considered minor edits. Thus, the plans with CTVs needing edits were scored as three. Physicians one and two scored six and two plans as three, respectively, which means replanning would be needed. Except for minor edits of the CTV, none of the plans required further modifications.

## 4 DISCUSSION

In this study, we developed, tested, and clinically evaluated an end‐to‐end solution that automatically generates VMAT plans for rectal cancers, given CT scan data and GTV. The evaluations by the radiation oncologists showed that the end‐to‐end solution could consistently contour the CTV and OAR, and could consistently produce clinically acceptable plans. All the necessary edits for either contours or plans were considered minor. None of the contours or plans were unusable. We report both quantitative and qualitative results, such as physician scoring and evaluations, to demonstrate the clinical acceptability of auto‐segmentation and auto‐planning processes individually and as a whole. By evaluating the quality of plans generated using contours from our auto‐segmentation method, our study goes beyond the conventional approach of solely comparing automated contours to manual contours. Instead, we aim to measure the clinical impact and practical applicability of the auto‐segmentation method holistically and more meaningfully via treatment plan quality based on physician reviews. The methodology and result of this study offers insights into how the automated contours directly influence the final treatment plans, and how they measure up against standards by different physicians. We found that automatically generated OAR contours were sufficient for auto‐treatment planning such that no replanning was required because of OAR contours. Predicted target volumes may require minor modifications and could result in automatic replanning. Future research should focus on improving CTV acceptability, particularly across various physicians.

For auto‐segmentation, previous studies have used more than 200 patient scans to train for OAR segmentations in rectal cancers on CT scans.^7^, ^15^ This study showed that high clinical acceptability of OAR contours can be obtained with a small but consistent and well‐curated training dataset, consistent with a recent finding using nnU‐Net.^13^ The automated contouring of large and small bowel can facilitate clinicians to employ separate dose objectives for different bowel structures, which often are too time‐consuming to contour without the aid of auto‐segmentation. The segmentation tool also provides a consistent sex‐specific contouring approach for genitalia that is not always observed in clinical practice.

In the auto‐segmentation, the model currently does not consider the use of vaginal dilators during patient simulations. Predictions on such CT scans that present drastic deviations in human anatomy compared to the training dataset should be carefully examined. There are considerable variations in how CTV contours are drawn and the extent to which they are clinically acceptable. Deployment of the tool would require consensus on the scoring and the behavior of the models. The use of an automated contouring tool may facilitate the development of consensus. Additionally, the inclusion of GTVp and GTVn as inputs for CTV segmentation was crucial to accurately define the superior and inferior extent of the auto‐generated CTV, particularly in cases with high or low‐lying tumors in the rectum. Excluding the GTV information would require significant modifications by physicians to the auto‐generated contour, rendering the segmentation tool less effective as part of the end‐to‐end system.

We compared the dosimetric values for the end‐to‐end plans against objectives employed in our clinics and the most recent consensus.^14^ The results showed that all plans passed the objectives except for one. All the plans that needed to be replanned were due to the predicted CTV contours, and not because of any issues with the performance of the auto‐planning algorithm. Based on these results, any future deployments of end‐to‐end solutions for treatment planning should investigate the discrepancies between the training data and the expectations of the end‐users, especially with regard to target volume contours.

A previous study used CNN to perform auto‐segmentation and dose predictions, from which DVH values were calculated and used in objective functions to perform dose calculations in the Pinnacle treatment planning system.^15^ The study achieved Dice coefficient (±SD) values of 0.87 ± 0.06 for CTV, 0.80 ± 0.11 for bladder, 0.79 ± 0.10 for left‐femoral head, and 0.80 ± 0.10 for right‐femoral head.^15^ Though it is challenging to directly compare without using the same testing dataset, our study achieved comparable Dice coefficient values for CTV and higher values for both bladder and femurs. Though the planning methodologies were not directly comparable, we improved on the clinical acceptability for the end‐to‐end testing compared with the previous study's 80% acceptability rate.^15^ Our study was validated with physician evaluations of not only the entire process but also the contouring and planning steps separately. The results of this study reaffirmed that the values for DVH objectives do not fully describe the clinical acceptability of a plan, and so physician evaluations are essential in understanding the clinical usage of a given automated tool.^16^

There were limitations in this study. First, we were unable to meaningfully compare the dosimetric results from the end‐to‐end plans with the clinical plans as the target volumes in the automatic plans were different from the clinical plans. This prevented us to use non‐inferiority statistical tests to compare the dosimetric results of the automatic plans versus the clinical plans. Second, the outcomes have only been tested and evaluated in our clinical practices following our guidelines. Variations in clinical practices exist. Further investigation, such as thorough performance testing and evaluations against local clinical practices, is required before safe deployment of the tool in other clinical settings. Third, different patient positioning (e.g., without belly board) may introduce limitations that have not been tested. Automated predictions on such scans should be discouraged or carefully scrutinized to ensure safety. Forth, we evaluated treatment plans for only short‐course radiotherapy and not conventional long‐course radiotherapy. However, our results are likely to be generalizable to long‐course radiotherapy.

## 5 CONCLUSION

This study demonstrated that the rectal cancer radiotherapy process for VMAT treatment planning can be automated to generate clinically acceptable and safe plans. Incorporating this algorithm into an automatic planning workflow can reduce planner burdens and improve planning efficiency.

## CONFLICT OF INTEREST STATEMENT

The authors have no relevant conflicts of interest to disclose.
